# Crosstalk Reduction Using a Dual Energy Window Scatter Correction in Compton Imaging

**DOI:** 10.3390/s20092453

**Published:** 2020-04-26

**Authors:** Makoto Sakai, Raj Kumar Parajuli, Yoshiki Kubota, Nobuteru Kubo, Mitsutaka Yamaguchi, Yuto Nagao, Naoki Kawachi, Mikiko Kikuchi, Kazuo Arakawa, Mutsumi Tashiro

**Affiliations:** 1 Graduate School of Medicine, Gunma University Heavy Ion Medical Center, 3-39-22 Showa-Machi, Maebashi 371-8511, Japan; 2 Department of Molecular Imaging and Theranostics, National Institute of Radiological Sciences, National Institutes for Quantum and Radiological Science and Technology, Anagawa 4-9-1, Inage, Chiba 263-8555, Japan; 3 Department of Radiation Oncology, Graduate School of Medicine, Gunma University, 3-39-22 Showa-Machi, Maebashi 371-8511, Japan; 4 Takasaki Advanced Radiation Research Institute, National Institutes for Quantum and Radiological Science and Technology, 1233 Watanuki-Machi, Takasaki 370-1292, Japan

**Keywords:** Compton camera, dual energy window, crosstalk reduction, simultaneous imaging, ^111^In, ^18^F

## Abstract

Compton cameras can simultaneously detect multi-isotopes; however, when simultaneous imaging is performed, crosstalk artifacts appear on the images obtained using a low-energy window. In conventional single-photon emission computed tomography, a dual energy window (DEW) subtraction method is used to reduce crosstalk. This study aimed to evaluate the effectiveness of employing the DEW technique to reduce crosstalk artifacts in Compton images obtained using low-energy windows. To this end, in this study, we compared reconstructed images obtained using either a photo-peak window or a scatter window by performing image subtraction based on the differences between the two images. Simulation calculations were performed to obtain the list data for the Compton camera using a 171 and a 511 keV point source. In the images reconstructed using these data, crosstalk artifacts were clearly observed in the images obtained using a 171 keV photo-peak energy window. In the images obtained using a scatter window (176–186 keV), only crosstalk artifacts were visible. The DEW method could eliminate the influence of high-energy sources on the images obtained with a photo-peak window, thereby improving quantitative capability. This was also observed when the DEW method was used on experimentally obtained images.

## 1. Introduction

**Figure 1:**
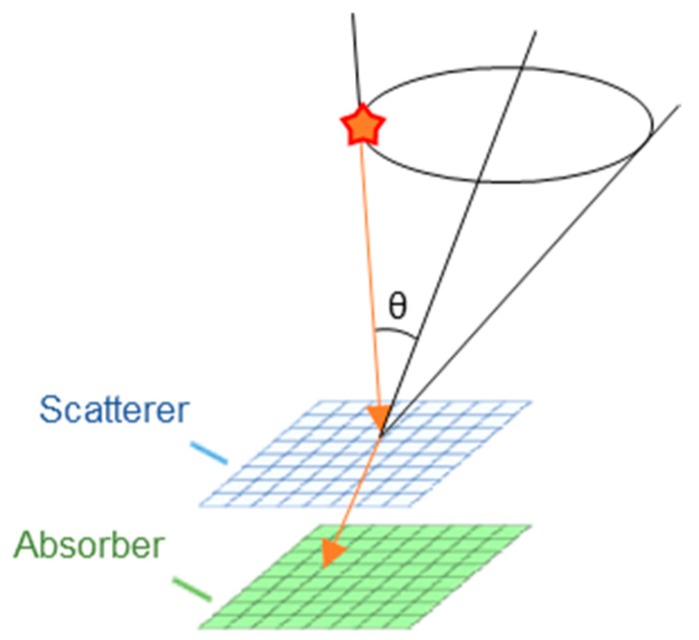
Schematic diagram of Compton imaging.

The Compton camera is the most promising device for the detection of gamma rays ranging from tens of keV to several MeV; it can identify the direction of gamma rays originating from radioisotopes based on the kinematics of Compton scattering. An elementary Compton camera consists of two types of position-sensitive detectors. The first detector (scatterer) detects a series of Compton scattering events; the second detector (absorber) detects absorption phenomena (Figure 1). Both detectors record the positions and deposited energies of the interaction (the Compton scattering and the photo-absorption). The scattered angle in the scatterer, *θ*, can be calculated from:

(1) $$\cos\;\theta =1-\frac{m_{e}c^{2}E_{1}}{E_{2}\left(E_{1}+E_{2}\right)},$$

where *m_e_c*^2^ is the rest mass of an electron, *E*_1_ is the energy loss of the incident photon in the scatterer, and *E*_2_ is the energy deposited in the absorber.

The Compton camera was originally developed in the field of astronomy [1,2]. However, in recent years, various types of Compton cameras have been proposed for medical use [3,4,5]. Compton cameras have several advantages in nuclear medicine and are consequently expected to be utilized as a new medical instrument for various tasks. In particular, Compton cameras can simultaneously image multi-tracers, including single-photon emission computed tomography (SPECT) and positron-emission tomography (PET) tracers [5,6,7,8,9,10]. Simultaneous imaging has the potential for clinical and molecular application in many areas [11,12,13,14]. For example, hypoxia would be observed more precisely if blood flow and hypoxia could be observed simultaneously [15]. Some reports have also indicated that multi-tracer SPECT is helpful in the differential diagnosis of parkinsonism [16]. When simultaneous imaging is performed, high-energy gamma rays sometimes scatter in the sample; some of them scatter in the first detector and scatter again in the second detector. In this case, they are detected as low-energy gamma rays. When the detected energy is accidentally in a lower energy window, crosstalk artifacts appear in the image. When this occurs, the detected energy is mistaken as an accumulation or overestimated [17,18]. Thus, crosstalk artifacts have to be removed for true analysis, because quantitative evaluation is important in nuclear diagnosis. Unfortunately, however, this aspect of crosstalk reduction in Compton imaging has never been studied.

In conventional SPECT, a dual energy window (DEW) subtraction method is used to reduce crosstalk artifacts [19,20]. In the DEW method, data are simultaneously acquired in two energy windows, a photo-peak window and a scatter window, to collect the crosstalk effect and remove the artifacts from the image. In this method, it is assumed that the number of events detected in the scatter window is correlated with the scattered component of the number of events detected in the photo-peak window. When the DEW method is used in SPECT imaging, the scattered component in the photo-peak window can be subtracted easily before reconstruction. The DEW method improves the image quality and quantitative performance [21,22]. In Compton imaging, however, list data (i.e., the energy and position data of each event) are required to reconstruct the image, and the crosstalk elements cannot be distinguished. Thus, in this study, we compared the reconstructed images produced using a photo-peak window and those produced using a scatter window. The two images were reconstructed separately and image subtraction was performed by identifying the differences between the two images.

## 2. Materials and Methods

### 2.1. Simulation Setup

**Figure 2:**
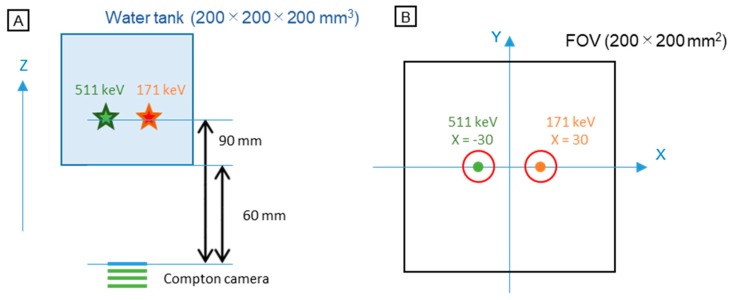
Schematic diagram of the simulation setup: (**a**) Lateral view; (**b**) top view. The red circles in (**b**) represent the region of interest (ROI) for quantitative analysis mentioned in Section 2.6. FOV: field of view.

To emulate our Compton camera, Monte Carlo simulation was performed using the GEANT4 toolkit. The simulation code is well-defined in previous studies [23,24,25]. A water phantom was created using a cube (200 × 200 × 200 mm^3^) and the Compton camera was placed under it at a distance of 60 mm from the bottom of the phantom. The direction perpendicular to the detector was set as the *z*-axis. Gamma rays of 171 and 511 keV—the energy of the gamma rays produced by ^111^In and ^18^F, respectively—were isotropically emitted from the locations (30, 0, 90) and (−30, 0, 90), respectively (Figure 2). The number of 171 keV gamma rays ranged from 1 × 10^8^–1 × 10^9^ and that of 511 keV gamma rays ranged from 1 × 10^9^–2 × 10^10^.

### 2.2. Compton Camera

Our Compton camera consisted of one layer of a silicon scatterer detector and three layers of cadmium telluride (CdTe) absorber detectors. The active area of each detector was 32 × 32 mm^2^. The thicknesses of the scatterer and absorber were 0.5 and 0.75 mm, respectively. The typical energy resolutions (full width at half-maximum (FWHM)) of the Si and CdTe detectors were 2.3 keV at 59.5 keV and 3.8 keV at 81.0 keV, respectively. The detectors were cooled to −20 °C to improve their energy resolution. The trigger threshold energy was set to 5 keV for all detectors. Further details are provided elsewhere [26].

### 2.3. Event Selection

In this study, only two-hit events in which incident photons simultaneously interacted with the Si detector and one of the CdTe detectors were investigated. Two-hit events whose total energies deposited in the scatterer and the absorber were 166–176 keV for the photo-peak of 171 keV (^111^In), 176–186 keV for the scatter window, and 506–516 keV for the photo-peak of 511 keV (^18^F) were selected. The energy windows were set based on the strength of the energy resolution of the Compton camera (the FWHM was approximately 6.8 keV for 171 keV gamma rays). The events that deposited 20–35 keV in the Si detector and also deposited the remaining energy in the top CdTe detector were eliminated because these are the typical energies of characteristic X-rays of cadmium and tellurium in the absorbers.

### 2.4. Image Reconstruction

The images were reconstructed using the backprojection (uniformly enlarged projection [27] with a Voigt function) method and the list-mode maximum-likelihood expectation-maximization (ML-EM) method [28]. In the backprojection of Compton imaging, a Compton cone is reconstructed from the vector joining two interaction points and the scattering angle (calculated from the Compton kinematics of Equation (1)). The pixel value *λ_j_* at the *j*th pixel can then be expressed as follows:

(2) $$\lambda_{j}=\underset{i}{\sum }V_{\left(\theta_{i};\sigma ,\gamma \right)},$$

where *i* is the event index, *V*($\theta_{i}$;*σ*,*γ*) represents the Voigt profile, *σ* and *γ* are the parameters of ARM determined by a point source imaging examination, and *θ_i_* is the minimum angular difference between the vector from the apex of the Compton cone to the reference point and the vector on the surface of the Compton cone of the *i*th event. The ML-EM algorithm is widely used for Compton image reconstruction, and the image is updated iteratively. For the *k*th iteration, the following step is calculated:

(3) $$\lambda_{j}^{\left(k+1\right)}=\frac{\lambda_{j}^{\left(k\right)}}{S_{j}}\underset{i}{\sum }\frac{t_{ij}}{\sum_{m}t_{im}\lambda_{m}^{\left(k\right)}},$$

where $\lambda_{j}^{\left(k\right)}$ is the pixel value in the image of the *k*th iteration, *S_j_* is the detection efficiency vector, and *t_ij_* is the transition probability of the *i*th event at the reference point.

The size of the field of view was 200 × 200 mm^2^, and the pixel size was 1 mm/px. The imaging plane was set at a distance of 90 mm from the scatterer. The number of ML-EM iterations was set to 30, which was decided by considering sufficient convergence and calculation time.

### 2.5. DEW

The data collected from the 171 and the 511 keV sources during Compton camera simulation were merged. A photo-peak window of 166–176 keV or a scatter window of 176–186 keV was applied to the merged data. After event selection, the images were reconstructed using either of the aforementioned energy windows and DEW images were produced by subtracting the scatter images from the photo-peak images via pixel-by-pixel calculation.

### 2.6. Quantitative Analysis

The integrated intensity in the region of interest (ROI) was calculated in the images with various combinations of 171 and 511 keV activities. ROIs were set around the point sources (red circles in Figure 2b); the radius was set to 15 mm. Two evaluation methods were employed. In the first method, the number of 171 keV gamma rays was fixed at 2 × 10^8^ and the number of 511 keV gamma rays was varied from 1 × 10^9^ to 2 × 10^10^. In the second method, the number of 171 keV gamma rays was varied from 1 × 10^8^ to 1 × 10^9^ and the number of 511 keV gamma rays was fixed at 1 × 10^10^.

### 2.7. Experimental Study

To evaluate the performance of the DEW method, a 17.3 MBq ^111^In point source (Indium(III) chloride, Nihon Medi-Physics) and a 1.7 MBq ^22^Na point source (SKR8252, Eckert & Ziegler) were placed in a cubic water tank above the Si/CdTe semiconductor Compton camera (^22^Na was used as a positron source). The diameter of each point source was approximately 2 mm. The distance from the Compton camera to the sources was 90 mm (same location as that in the simulation study). The Compton camera consisted of a double-sided strip Si detector and three CdTe detectors. Further details are provided elsewhere [26]. Measurement was carried out for five hours and one hour for ^111^In and ^22^Na, respectively. The DEW images were reconstructed in the same manner as that described in Section 2.5.

## 3. Results

**Figure 3:**
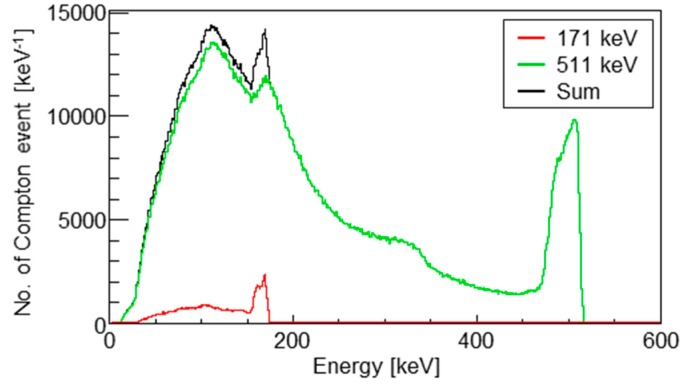
Energy spectra of the detected photons from the 171 keV source (red), the 511 keV source (green), and the summation of them (black). The numbers of generated 171 and 511 keV gamma rays were 2 × 10^8^ and 1 × 10^10^, respectively.

Figure 3 shows the energy spectra of the 171 and 511 keV point sources obtained via simulation calculations; 2 × 10^8^ and 1 × 10^10^ gamma rays were generated, respectively. Scattered photons of 511 keV gamma rays were observed in the photo-peak window of 171 keV. Thus, a large number of scattered events were involved in the photo-peak window of 171 keV.

**Figure 4:**
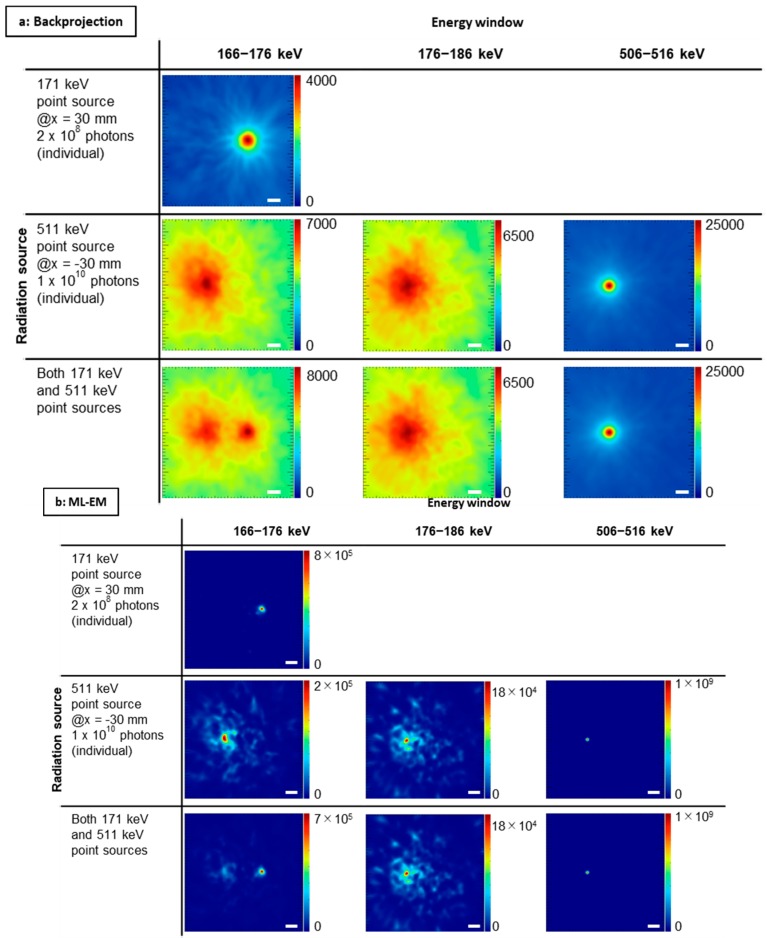
Reconstructed images for each combination of source energy and energy window condition: (**a**) Backprojection; (**b**) maximum-likelihood expectation-maximization (ML-EM). White bar = 2 cm. The color scale represents an arbitrary unit.

Figure 4 shows the reconstructed images for each combination of source energy and energy window condition using the data of Figure 3. The color scale used represents an arbitrary unit—that is, the scale correlates with radiation activity but was not calibrated. The numbers of generated 171 and 511 keV gamma rays were 2 × 10^8^ and 1 × 10^10^, respectively. Crosstalk artifacts were present in the images obtained using the 511 keV point source data (middle and lower lines in Figure 4).

**Figure 5:**
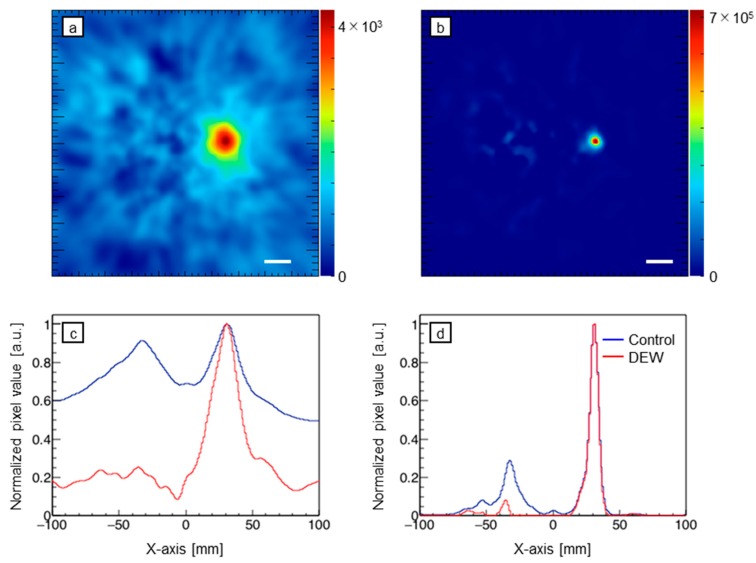
Dual energy window (DEW) images obtained using backprojection and ML-EM images: (**a**) Backprojection; (**b**) ML-EM; (**c**) and (**d**) corresponding normalized profiles of x-projection at y = 0 of (**a**) and (**b**), respectively (red lines), compared with those of control images (blue lines). White bar = 2 cm. The color scale represents an arbitrary unit.

**Figure 6:**
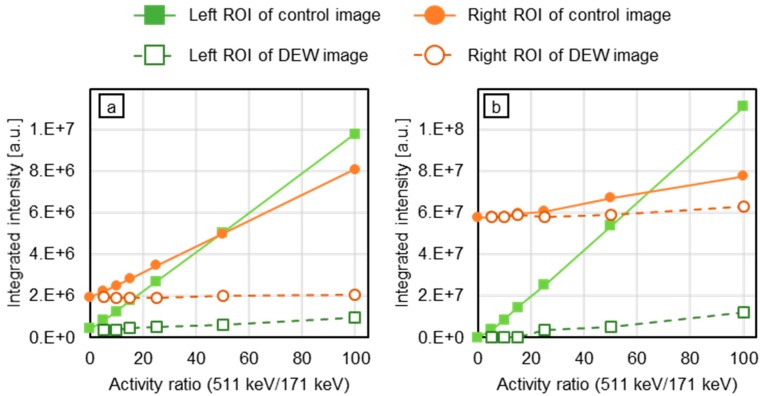
Integrated intensity in the ROI: (**a**) Backprojection; (**b**) ML-EM. The number of 171 keV gamma rays (generated in the right ROI) was fixed, and the number of 511 keV gamma rays (generated in the left ROI) was varied from 0 to 100 times the number of 171 keV gamma rays. The *x*-axis represents the ratio of number of generated 511 keV gamma rays to number of generated 171 keV gamma rays.

**Figure 7:**
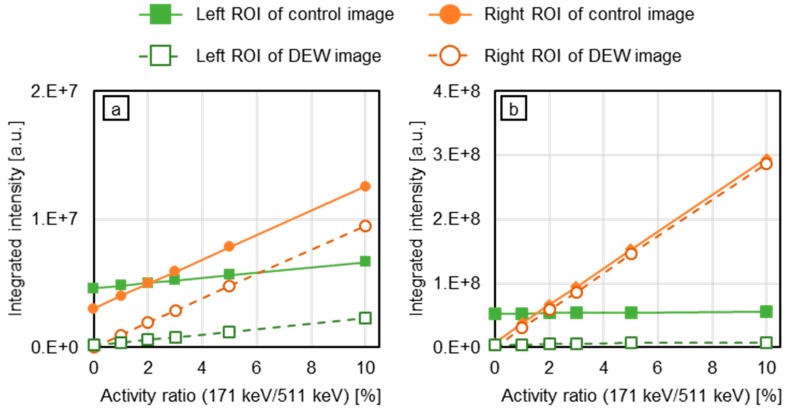
Integrated intensity in the ROI: (**a**) Backprojection; (**b**) ML-EM. The number of 511 keV gamma rays (generated in the left ROI) was fixed and the number of 171 keV gamma rays (generated in the right ROI) was varied from 0% to 10% the number of 511 keV gamma rays. The *x*-axis represents the ratio of number of generated 171 keV gamma rays to number of generated 511 keV gamma rays.

Using the images of Figure 4, DEW images were obtained (Figure 5). Compared with the control images (the lowest row of Figure 4a,b), the crosstalk is considerably suppressed. In the DEW image of backprojection, the FWHM is smaller. Figure 6 and Figure 7 represent the integration of pixel values in the ROIs. In Figure 6, the number of gamma rays of 171 keV generated was fixed and that of 511 keV was increased from zero to 100 times that of 171 keV. By contrast, the activity of 511 keV was fixed and that of 171 keV was varied from 0% to 10% that of 511 keV (please note that the numerator and the denominator of the fractions are reversed). In both cases, the crosstalk artifacts were suppressed in the DEW images.

**Figure 8:**
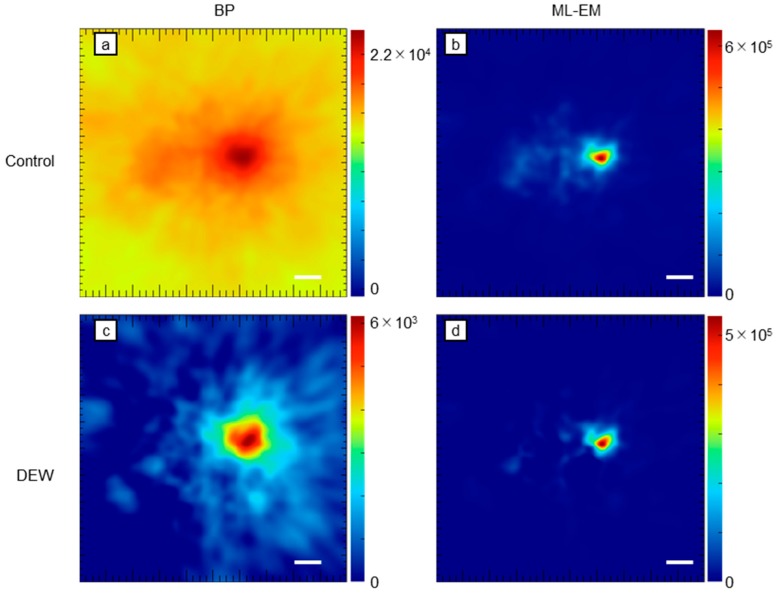
Control and DEW images obtained via an experimental study: (**a**) Control backprojection (BP) image; (**b**) control ML-EM image; (**c**) DEW BP image; (**d**) DEW ML-EM image. White bar = 2 cm. The color scale represents an arbitrary unit.

Finally, we determined the efficacy of the DEW method by conducting an experimental study (Figure 8). The DEW method could also successfully suppress the crosstalk and hence is effective in experimental studies as well.

## 4. Discussion

Multi-tracer imaging is still at an early stage of development; however, it has the potential for clinical and molecular application in many areas [11,12,13,14]. In simultaneous imaging, crosstalk artifacts reduce the power of the test and quantitative capability [17,18]. The DEW method is a scattering correction technique for SPECT and can be also applied to reduce crosstalk in simultaneous imaging [20]. If crosstalk artifacts could be suppressed, the dose of tracer would be reduced, and Compton cameras with fewer requirements could be used.

In this study, we performed simultaneous imaging with 171 and 511 keV gamma-ray sources; gamma rays of 171 keV are emitted from ^111^In, which is one of the most commonly used isotopes for SPECT, and 511 keV is the annihilation energy of the gamma rays used for positron-emission tomography (PET). As the backscatter energy of 511 keV gamma rays is 170 keV, crosstalk is significant in this combination (Figure 3).

In the images obtained using a photo-peak energy window of 171 keV, presented in Figure 4, there are recognizable crosstalk artifacts of 511 keV gamma rays. However, in the DEW images (Figure 5), these crosstalk artifacts have disappeared. To perform quantitative analysis, we evaluated the pixel value around the source position (Figure 6 and Figure 7). Referring to Figure 6, the activity of the 171 keV source was fixed, and the activity of the 511 keV source was varied from zero to 100 times that of the 171 keV source. The integrated intensity for the left ROI should be zero; however, the pixels on the left ROI present a small intensity according to the point spread function of the 171 keV point source image. Regardless of the case, the intensity must be constant and not be affected by the 511 keV source activity. However, the pixel values of the control images were proportional to the activity of the 511 keV source (solid green line in Figure 6). By means of the DEW method, the influence of the 511 keV source activity in the left ROI is reduced (the gradient of the dashed green line is smaller than that of the solid green line). On the other hand, the integrated intensity of the right ROI was supposed to be constant if the 511 keV had no impact. The DEW method eliminates the influence of the 511 keV source and reduces it to a proper value. In the ML-EM images, the ratio of integrated intensity of the left ROI to that of the right ROI is small because the point spread function (PSF) is smaller than that of backprojection. In Figure 7, the number of generated gamma rays of 511 keV was fixed, and that of 171 keV was varied from 0% to 10% the number of generated 511 keV gamma rays. Under this condition, crosstalk was suppressed (green lines), and the integrated values in the right ROI (solid orange lines in Figure 6) were proportional to the activity of the 171 keV source (the constant component was reduced to approximately zero); this was particularly observed in the backprojection images.

In this study, the DEW method could not completely remove the crosstalk effect from the images. This occurred because the number of detected events from the 511 keV primary photons in the photo-peak window (166–176 keV) was larger than that in the scatter window (176–186 keV). From the detected spectrum of the 511 keV source, the ratio of the number of events in the photo-peak window to that of the scatter window was 106%. In the DEW method for SPECT imaging, the crosstalk component in the photo-peak window is assumed from the scatter window component and corrected by a factor *k* [29]; a *k* value of 106% would reduce crosstalk artifacts effectively. The appropriate value of *k* depends on the source distribution, shape of the subject, and the primary source energy [30,31]. Thus, *k* should be estimated from the gradient of the spectra or simulation calculations [32,33,34]. An adequate *k*-value estimation method should be investigated in future work. As an alternative approach, the triple energy window (TEW) method, which employs a main photo-peak window and two sub-windows (an upper sub-window and a lower sub-window), can be used. When the energy spectrum is uniform around the photo-peak energy window, depending on the combination of probe radioisotopes (RIs), the TEW method can be applied, and *k*-value estimation should not be required [35,36].

There are also many scattered events around 110 keV in the spectra of the 511 keV source (green line in Figure 3). Thus, crosstalk artifacts would appear not only for the combination of ^18^F and ^111^In, but also for that of ^18^F and ^99m^Tc (141 keV). The number of scattered gamma rays increases with the depth of the accumulation position. In this study, the water depth of the point source was 30 mm. If the depth is increased, the number of scattered events may increase, the photo-peak signal may decrease in the opposite direction, and crosstalk artifacts could present a significantly greater impact on the image.

In our previous study, the crosstalk effect on the Compton camera was smaller than that on conventional SPECT images [6]. It is assumed that one of the reasons for this phenomenon is that the energy resolution of the Compton camera is superior to that of the conventional detector (scintillation camera). Thus, crosstalk is expected to be significant on images taken by a Compton camera with low-energy resolution.

The crosstalk artifacts appeared blurred around the 511 keV point source (Figure 4). In the case of a source with complex distribution, crosstalk artifacts would be produced similarly. Thus, the DEW methods would be applied to sources with complex distributions. Although it is difficult to confirm every shape and combination of sources, naturally some distributed sources (e.g., the Shepp–Logan phantom and the National Electrical Manufacturers Association (NEMA) phantom) should be confirmed in future studies. The DEW method can easily calculate the difference of two images without applying an excessive load onto a computer, although the load on the computer could be large when a Compton image of a distributed source is reconstructed with the ML-EM method.

Compton imaging is applied to range estimation in particle therapy [37,38,39] and to environmental measurements [40,41,42]. In these applications, the scattered noise component is larger than that in nuclear medicine imaging, because the number of high-energy radiations significantly exceeds that of signals radiations. The DEW method could also improve the quantitative analyses performed in these fields.

In summary, the DEW scatter correction method could be performed via pixel-by-pixel subtraction, can suppress crosstalk artifacts, and can improve the quantitative capability of Compton imaging.
